# Effect of 5-Aminolevulinic Acid Photodynamic Therapy on *Aspergillus fumigatus* Biofilms in Vitro

**DOI:** 10.1007/s00284-023-03351-8

**Published:** 2023-09-02

**Authors:** Zhimin Duan, Jianbo Tong, Nana Zheng, Rong Zeng, Yuzhen Liu, Min Li

**Affiliations:** 1 Department of Dermatology, Nanjing Jiangning Hospital, Nanjing, 211100 Jiangsu China; 2 Hospital for Skin Diseases (Institute of Dermatology), Jiangsu Key Laboratory of Molecular Biology for Skin Diseases and STIs, Chinese Academy of Medical Sciences and Peking Union Medical College, Nanjing, 210042 Jiangsu China; 3 Department of Dermatology, Institute of Dermatology, Jiangxi Academy of Clinical Medical Sciences, The First Affiliated Hospital of Nanchang University, No. 17 Yongwaizheng Street, Nanchang, 330001 Jiangxi China; 4 Center for Global Health, School of Public Health, Nanjing Medical University, Nanjing, 211166 Jiangsu China; 5 Department of Dermatology, The First Affiliated Hospital of Yunnan Traditional Chinese Medicine University, No. 120 Guanghua Rd, Kuming, 650021 China

## Abstract

*Aspergillus fumigatus* biofilm development results in enhanced pathogenicity and treatment resistance. Most contemporary antibiotics, however, are unable to eliminate biofilms. In recent years, with the application of new photosensitizers and the development of treatment, ALA-PDT (5-aminolevulinic acid photodynamic treatment) has achieved remarkable curative effect in the treatment of fungal infectious diseases; however, no research has been conducted on ALA-PDT against *A. fumigatus*. This study investigated the inhibitory effect of ALA-PDT at various 5-aminolevulinic acid concentrations and light doses on *A. fumigatus* planktonic and biofilms in vitro. We found that ALA-PDT may successfully inhibit the development of *A. fumigatus* biofilm and disintegrate mature biofilm. After ALA-PDT treatment, the adherence rate and vitality dramatically decreased, and the biofilm’s structure was severely compromised. Our findings show for the first time that ALA-PDT may be used to prevent the formation of *A. fumigatus* biofilm and disturb the structure of mature biofilm, and that it could be employed as a therapeutic therapy for *A. fumigatus* superficial infection.

**Supplementary Information:**

The online version contains supplementary material available at 10.1007/s00284-023-03351-8.

## Introduction

In recent years, the prevalence of primary cutaneous aspergillosis (PCA) has risen dramatically. According to studies, PCA was found in 43 percent of children who died of invasive aspergillosis [1]. *A. fumigatus* is the most frequent aspergillosis strain, which can cause clinically deadly infectious illnesses [2–4], and it exhibits resistance to a number of standard antifungal medicines used in clinic during therapy [5]. Drug resistance may be linked to the establishment of an *A. fumigatus* biofilm, which has considerably higher antifungal resistance than planktonic cells [6–9]. Simply raising the dosage of antifungal medicines will not solve the problem of drug resistance, but may exacerbate the drugs' negative effects. As a result, unique and effective therapies that are distinct from those provided by traditional pharmaceuticals are urgently needed.

Photodynamic therapy (PDT) has been suggested as a viable treatment for microbial infection [10–13]. A photosensitizer, visible light for irradiation therapy, and tissue oxygen are the three main components [12]. Warts [14, 15], acne vulgaris [16–18], infectious granuloma [19], and infective ulcer [20] are among the skin illnesses linked with microbial infections that 5-aminolevulinic acid photodynamic treatment (ALA-PDT) is thought to be beneficial in treating. ALA is a prodrug that may be taken by active cells, then activated by a light-emitting diode (LED) and transformed into protoporphyrin IX, a natural photosensitizer. Protoporphyrin IX causes a photosensitive reaction that releases singlet oxygen and free radicals to kill target cells when illuminated at the right wavelength [12].

The effects of ALA-PDT on *Staphylococcus aureus* [21–24], *Pseudomonas aeruginosa* [25, 26], and *Candida albicans* [27] have been studied, and the results show that ALA-PDT has a potent antibacterial impact on these pathogenic bacteria. However, research on ALA-PDT's effectiveness against *A. fumigatus* and associated biofilms is currently restricted. As a result, the goal of our study is to see if ALA-PDT can suppress the production of *A. fumigatus* biofilm and remove mature biofilm. To our knowledge, this is the first study to investigate the effects of ALA-PDT on *A. fumigatus* biofilms.

## Materials and Methods

### Strains and Conidial Preparation

The reference strain utilized in the research was *A. fumigatus* Af293 (ATCC MYA-4609, CBS101355), which was isolated from a lung biopsy taken from a patient who ultimately died from invasive aspergillosis. In Supplementary Figs. 2, 3 *A. fumigatus* clinical strains (A1f, 02436, 02439) were used. The 3 *A. fumigatus* were isolated from sputum and bronchoalveolar of patients in Tongji Hospital of Tongji University and stored at Chinese Academy of Medical Sciences Collection Center of Pathogen Microorganisms-D (CAMS-CCPM-D). At 4 °C, the isolate was kept on Sabouraud dextrose agar (SDA) gradients. To make the biofilm, the conidia suspension needs to be prepared. To confirm the purity and viability, isolates was cultured on an SDA plate at 37 °C for 72 h. According to a previously established procedure [6, 28], a cell suspension of 1 × 10^5^ conidia ml^−1^ was made in RPMI 1640 media with 0.025 percent Tween-20. All *A. fumigatus* operations were carried out in a biosafety cabinet. Throughout the tests, a super-clean bench in the laboratory was utilized to manipulate *A. fumigatus*.

### Biofilm Formation

Biofilm generation methods for *A. fumigatus* have previously been reported [28]. A biofilm was formed on a coverslip in a 24-well tissue culture plate with a flat bottom (Corning Incorporated, New York, USA). A cover slip was placed on a 24-well tissue culture plate in advance, which may be employed for biofilm attachment and growth. Then 50 μL of the aforesaid conidial solution was seeded onto 24-well cell culture plates. A confocal laser scanning microscope (CLSM) was used to examine the formation of biofilm formations. XTT tests were used to determine the vitality of biofilm at time periods of 4 h, 8 h, 10 h, 16 h, 24 h and 48 h.

### CLSM Analysis of Biofilms

The pictures of the biofilms were captured using CLSM (Olympus FV1000). According to the manufacturer’s directions, the biofilms were stained with FUN-1 (Molecular Probes, Eugene, Oregon), which can bind the intravacuolar structures of the fungal cells. FUN-1 solution was applied to the surface of biofilms at a concentration of 25 mol/L (200 μL), then incubated at 37 °C for 20 min in the dark. To further observe the biofilms structure, polysaccharides of extracellular matrix were stained with Concanavalin A Alexa fluor 488 (CAAF, 25 μg/mL), and cell wall was stained with Calcofluor white (CFW, 25 μM), then incubated for 30 min at 37 °C in the dark. The biofilms’ surfaces were then cleaned with phosphate-buffered saline (PBS) and CLSM was used to examine them. The biofilms were seen at a magnification of 200 using a laser with an excitation wavelength of 488 nm. Conidia concentrations were measured early in the biofilm development process (4 h), and biofilm thickness was measured afterwards. CLSM scanned the biofilm thickness layer by layer from top to bottom at 1 m intervals. 3D Olympus Fluoview software was used to create 3D pictures of biofilms.

### XTT Reduction Assay

The XTT reduction test was used to assess the vitality of the *A. fumigatus* biofilms. For each experiment, the XTT/menadione reagent (Sigma-Aldrich, USA) was newly produced. The XTT/menadione solution was made by dissolving 2 mg of XTT in 10 mL of PBS, then adding 100 mL of a 10 mM menadione stock solution (0.4 mM in acetone). Following the incubation of the *A. fumigatus* conidia suspension for testing, each well of the 96 multi-well plate was filled with 100 μL XTT/menadione solution and incubated in the dark for 3 h at 37 °C. After incubation, the absorbance was measured using a microplate reader at a wavelength of 490 nm.

### ALA-PDT Treatment

To generate a 500 mM stock solution, ALA (Fudan-Zhangjiang Biopharmaceutical, Shanghai, China) was diluted in PBS. For disinfection, the solution was filtered through a 0.22 μm filter and kept at 4 °C in the dark. The solution was made from scratch and used within 10 min. The toxic effects of ALA or LED alone on mature biofilms were first explored. After the mature biofilms was successfully constructed, the ALA supernatant (5 mM, 15 mM and 30 mM) was incubated for 2 h or placed directly in the LED (50 J/cm^2^, 100 J/cm^2^ and 200 J/cm^2^), and then the vitality changes of the mature biofilms were detected using the XTT method. We then explored the optimal ALA drug concentration and incubation time for ALA-PDT. The mature biofilms were supplemented with varying amounts of ALA (5, 10, 15, 20 and 30 mM) and varying incubation time (0.5 h, 1 h, 2 h and 4 h) to explore the effects of ALA. For the follow-up investigation, a concentration of 15 mM and an incubation time of 2 h were used.

The excitation source for ALA was a light-emitting diode (LED) array with a wavelength of 635 nm (Lingyun photoelectronic system, Wuhan, China). The light irradiation intensity was 100 mW/cm^2^, and the fluences were set to 50 J/cm^2^, 100 J/cm^2^ and 200 J/cm^2^.

The planktonic cells and biofilms were treated with ALA-PDT by administering 15 mM ALA and exposing them to LED light. Control (ALA-LED-), ALA alone (ALA + LED-), LED alone (ALA-LED +), ALA-PDT1 (ALA + LED [50 J/cm^2^]), ALA-PDT2 (ALA + LED [100 J/cm^2^]), and ALA-PDT3 (ALA + LED [200 J/cm^2^]) were the six experimental groups. The samples were first incubated for 20 min in an ALA solution at 37 °C in the dark. The cells were then exposed to LED light.

### Intervention with ALA-PDT During the Early Stages of Biofilm Development

For biofilm development, 500 μL or 100 μL prepared cell suspension (1 × 10^5^ conidia ml^−1^) was applied to the 24/96-well plate in triplicate. The early stage of biofilms was treated as described above after 4 h of incubation at 37 °C. In a 24-well plate, samples on cover slips were washed three times with PBS after being treated with ALA-PDT. Using a CLSM, confocal images were acquired after staining with FUN-1. For data analysis, five fields of view were counted and the average value of the cells was taken. Samples in a 96-well plate were washed three times with PBS before being evaluated for biofilm viability using the XTT assay. 100 µL of XTT/menadione solution was added to the sample, which was then incubated at 37 °C for 3 h in the dark before being measured at 490 nm. Each test was examined in at least three different ways.

### Intervention with ALA-PDT During the Maturity of Biofilm Formation

The maturation stage of biofilms was treated as described above after 24 h of incubation at 37 °C. As indicated above, mature biofilms on cover slips in 24-well plates were dyed with FUN-1 and analyzed using CLSM. The thickness of the biofilm was measured at five separate locations on the cover slips, and the average value was calculated. The XTT test was used to determine the vitality of a mature biofilm in a 96-well plate, as described above. All experiments were carried out three times in total, with at least three duplicates each time.

### Strains Identification

The isolates were identified strictly by molecular sequencing of the ITS gene and small subunit ribosomal RNA gene. Mycelia of isolates were collected from SDA plates. According the manufacturer’s instructions, DNA extractions were performed by using the commercial EZNA Fungal DNA Mini Kit (D3390, Omega bio-teck, Doraville, USA). PCR reactions using the universal primers ITS1/ITS4 were performed in 20 µL as a final volume, containing 100 ng of DNA, 10 µL of 2 × PCR Master Mix and 1 µM of each primer. The primer sequence of ITS1 is 5′- TCCGTAGGTGAACCTGCGG-3′, and the primer sequence of ITS4 is 5′- TCCTCCGCTTATTGATATGC-3′. The primer sequence of NS1 is 5′- GTAGTCATATGCTTGTCTC-3′, and the primer sequence of NS4 is 5′- CTTCCGTCAATTCCTTTAAG-3′. Sequences were subjected to blast in NCBI database and strain information were obtained.

### Sequencing Data

The GenBank accession numbers for sequences of ITS of clinical isolates A1f, 02436 and 02439 are respectively OP630573, OP630595 and OP630596. The GenBank accession numbers for sequences of small subunit ribosomal RNA of isolates A1f, 02436 and 02439 are respectively OQ978672, OQ978845 and OQ978830.

### Statistical Analysis

A minimum of three separate experiments were carried out in each case. The average and standard deviation are used to show the data (S.D.). The comparison between three or more groups was done using one-way analysis of variance (ANOVA). A significant difference was defined as a p-value of less than 0.05 (*p* < 0.05).

## Results

### Biofilm Formation in *A. fumigatus*

**Fig. 1:**
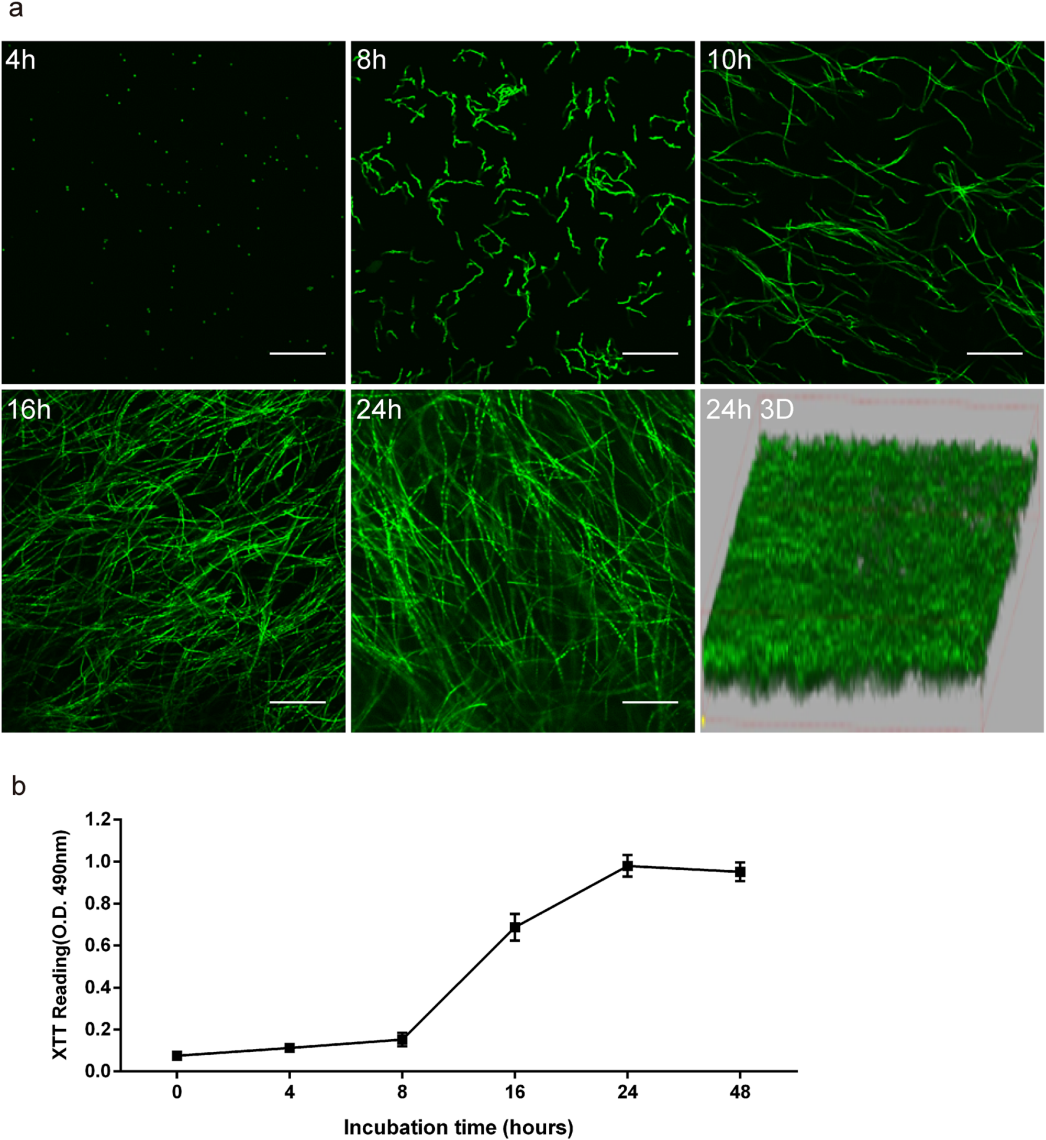
The formation of *A. fumigatus* biofilms and the changes in viability that occur throughout this process. Confocal laser scanning microscopy (CLSM) was used to capture images of *A. fumigatus* biofilms. The biofilms were photographed at five different time intervals: 4 h, 8 h, 10 h, 16 h, and 24 h). Software was used to create a three-dimensional picture of the biofilm at 24 h. FUN-1 was used to dye the cells. The thickness of the biofilm is depicted in three-dimensional photographs. The scale bar symbolizes 100 μm, and the original magnification was × 200. The XTT test revealed variations in viability during biofilm development (**b**)

*A. fumigatus* biofilm was effectively formed in vitro, and CLSM was used to record the overall process of biofilm development, as shown in Fig. 1a. At 4 h, the conidia began to cling to the coverslip, and by 8 h, they had all fully budded to produce hyphae. At 10 h, the mycelia were found to develop perpendicular to the slide’s growth direction, forming a rudimentary three-dimensional structure. At 16 h, the 3D structure was significantly strengthened, and at 24 h, it had achieved its most mature state. Using confocal microscopy software, the 3D morphology was recreated. During the creation phase, the vitality of the biofilm steadily increased (Fig. 1b). After the first 10 h of culture, its viability rose marginally. The biofilm activity considerably enhanced over the next 10 to 24 h of culture. However, within 24 h to 48 h of culture, it reached a plateau, indicating that mature biofilms have already formed. Biofilms were stained with CAAF combined with CFW. CFW labels cell wall structure and Concanavalin A shows the production of extracellular polysaccharides from biofilms (Supplementary Fig. 1).

### The Effect of Single ALA on Mature Biofilms

**Fig. 2:**
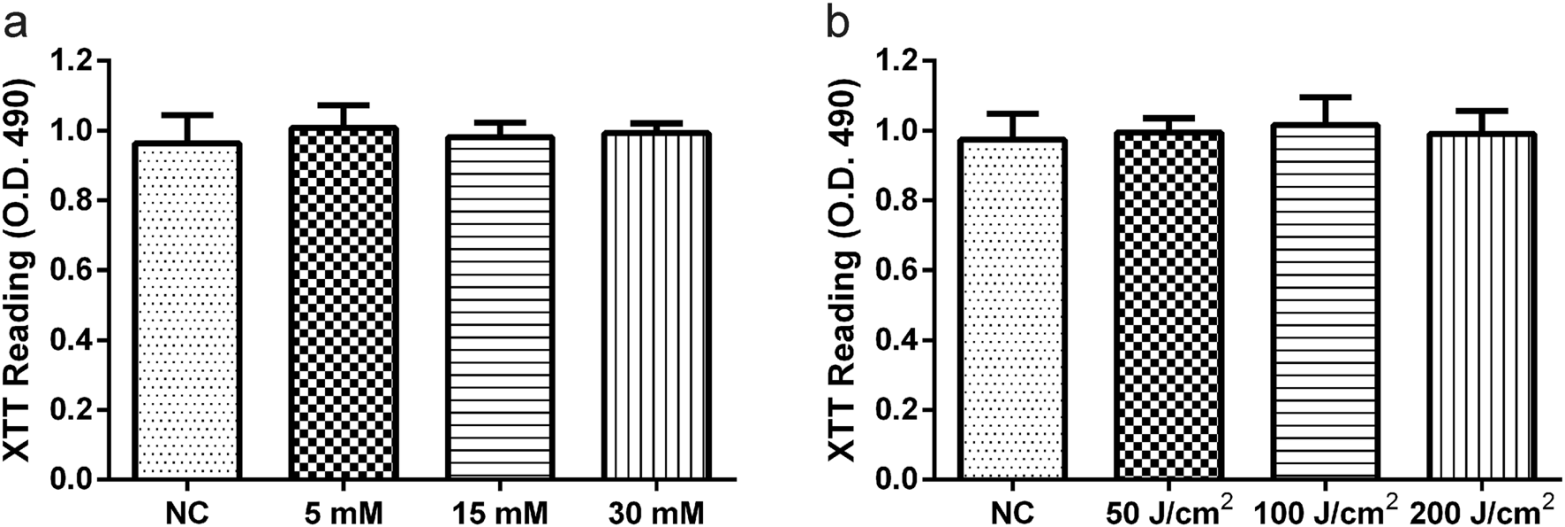
*A. fumigatus* biofilms treated with ALA alone (15 mM, 20 mM, 30 mM) (**a**) or LED alone (50 J/cm^2^, 100 J/cm^2^, 200 J/cm^2^) (**b**) in an XTT experiment. The metabolic activity of the cells did not differ substantially amongst the groups studied

We used the XTT test to compare the ALA-treated *A. fumigatus* mature biofilms to the control biofilms to see if ALA is harmful to mature biofilms (Fig. 2a). For 24 h, mature biofilms were given three different doses of ALA (5 mM, 15 mM and 30 mM). The three ALA concentration groups’ OD values were not statistically different from the control group’s (*p* > 0.05).

### The Effect of Single LED on Mature Biofilms

To see if LED alone has an effect on biofilm survivability, we treated mature biofilms with three different light intensities (50, 100 and 200 J/cm^2^) (Fig. 2b). Between the three energy groups and the control group, there was no significant difference in biofilm viability. In either of the light irradiation groups, there was no toxicity (*p* > 0.05).

### The Effect of Different Concentration and Incubation Time of ALA on Mature Biofilms

**Fig. 3:**
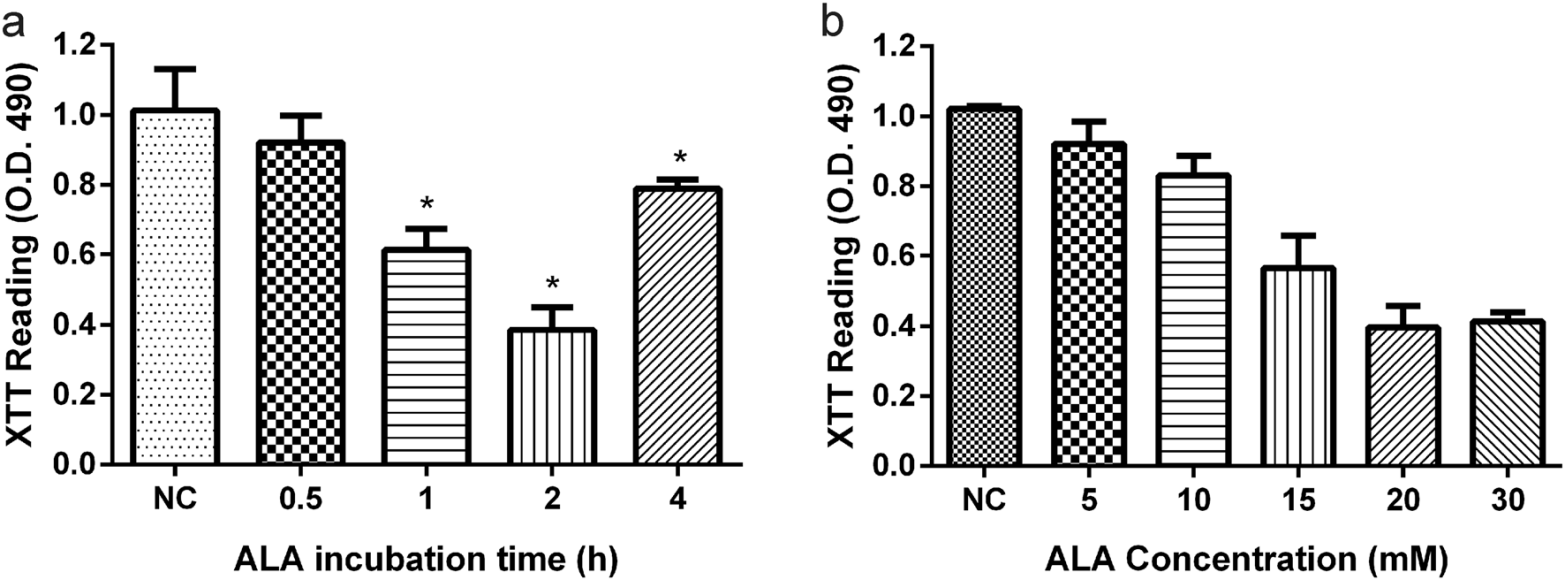
*A. fumigatus* biofilms treated with ALA and LED in an XTT experiment, and we used different incubation times (**a**) and concentrations (**b**) of ALA to explore the optimal treatment conditions. * *p* < 0.05 is a significant difference

In order to explore the best concentration and incubation time of ALA on mature biofilms, the mature biofilms were supplemented with varying amounts of ALA (5 mM, 10 mM, 15 mM, 20 mM and 30 mM) and varying incubation times (0.5 h, 1 h, 2 h and 4 h) to explore the best treatment conditions of ALA (Fig. 3). For the follow-up investigation, a concentration of 15 mM and an incubation time of 2 h were used.

### Effects of ALA-PDT on Biofilm Morphology and Vitality

**Fig. 4:**
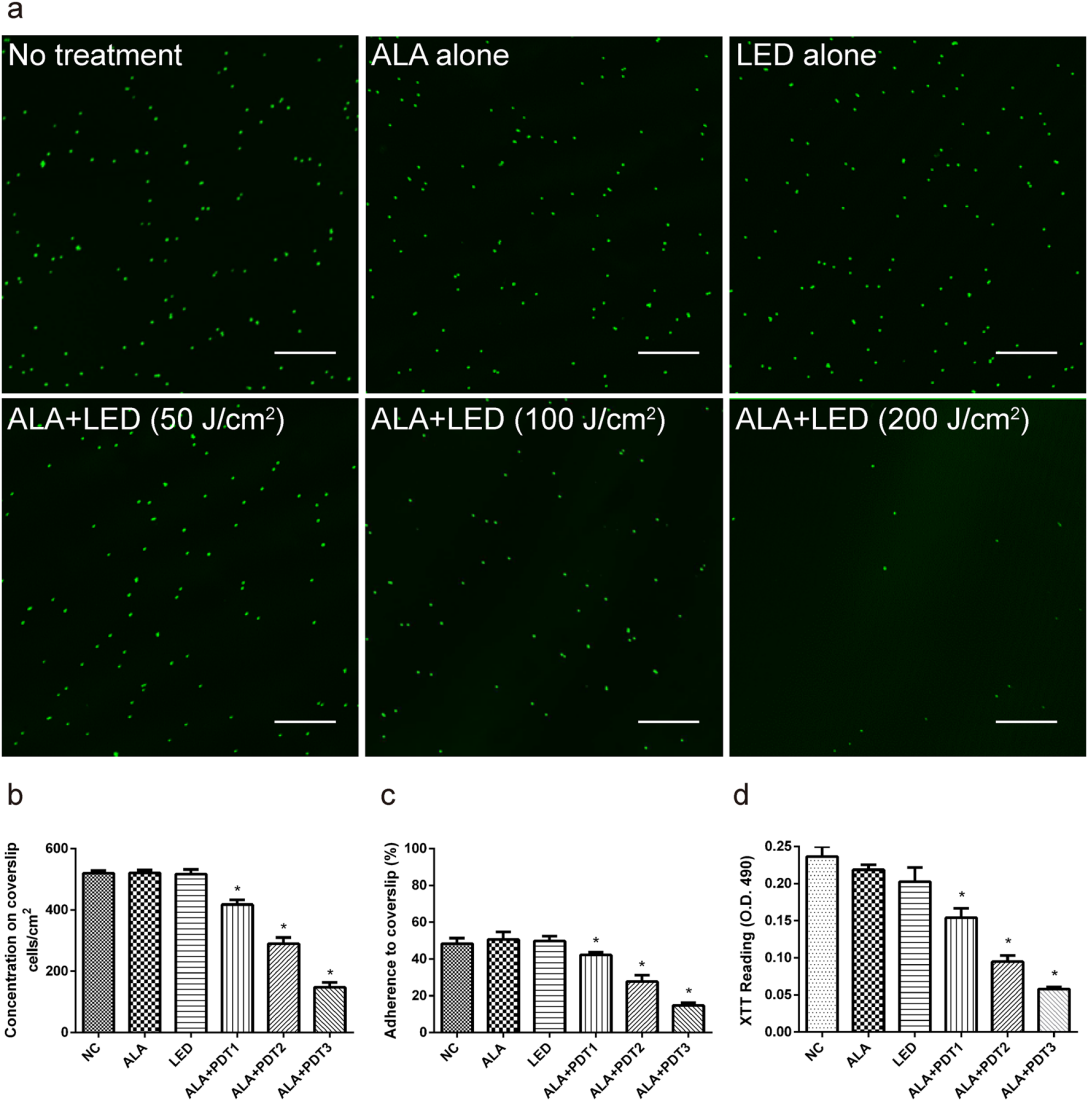
Changes in adhesion levels in the early stages of biofilm development following various ALA-PDT treatments (4 h). CLSM photographed and studied the biofilms. The morphological alterations of *A. fumigatus* biofilms treated with various ALA-PDT were shown in Fig. 4a photos. The figure on the left top depicted the no-treatment group. ALA alone, LED alone, ALA-PDT1 (ALA + LED [50 J/cm^2^]), ALA-PDT2 (ALA + LED [100 J/cm^2^]), and ALA-PDT3 (ALA + LED [200 J/cm^2^]) were used to cure *A. fumigatus* biofilms. **b** CLSM used the following calculation to compute the cell concentration on the coverslips depending on the number of cells: The ratio of the number of cells in the imaged region to the size of the area. The ALA-PDT group had a lower level of toxicity than the control group. **c** The following formula was used to normalize adherence rates with the cell number measured by CLSM: The ratio of the number of cells adherent to coverslips to the total number of cells injected in the test well. **d** The ALA-PDT treatment's inhibitory efficacy on early phase of *A. fumigatus* biofilm development was determined using the XTT test. The OD value of the control group was substantially greater than that of the ALA-PDT groups. * *p* < 0.05 is a significant difference. Scale bar is 100 μm

**Fig. 5:**
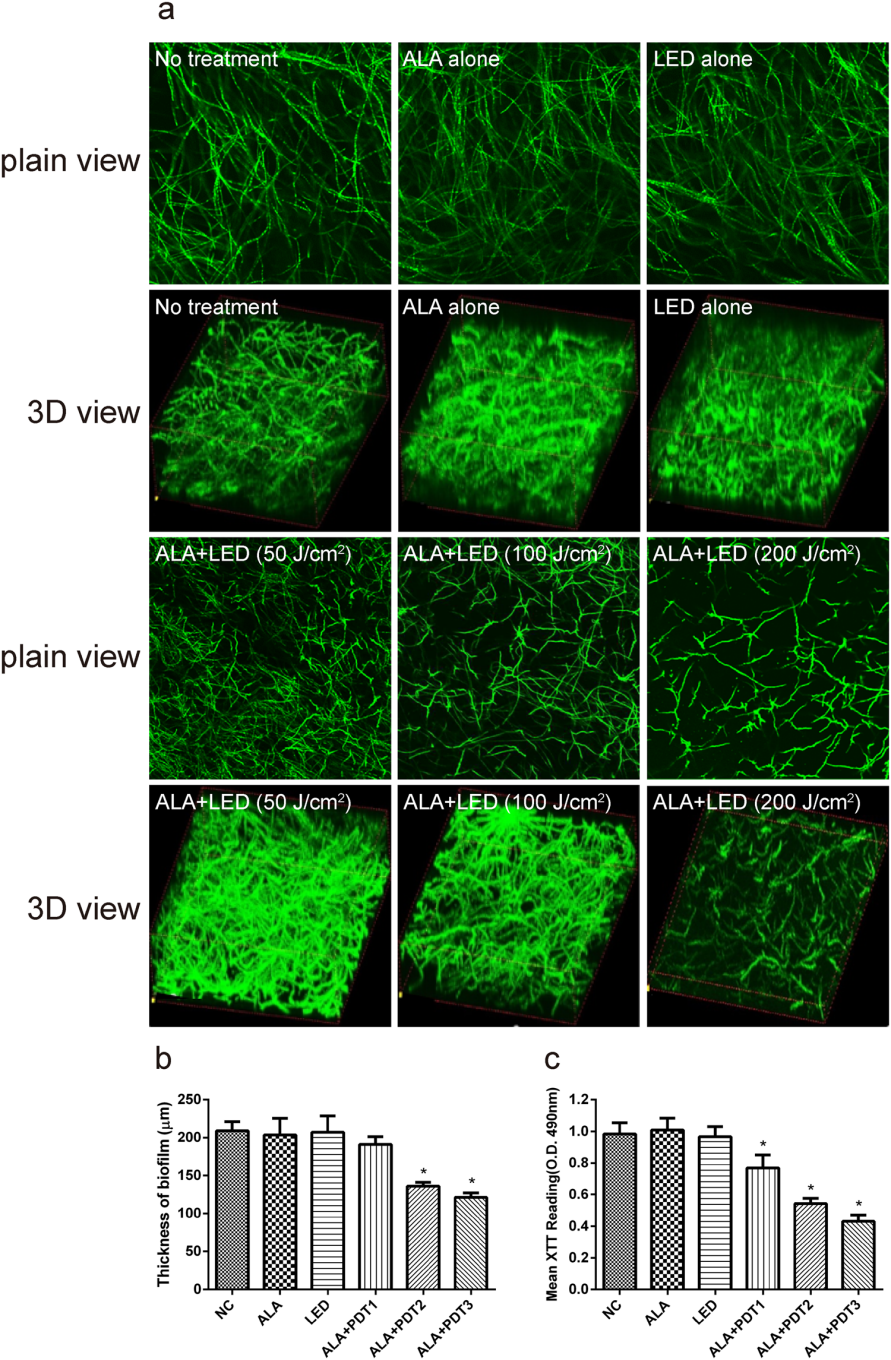
After varied ALA-PDT treatments at the late stage of biofilm development, changes in biofilm morphology, thickness, and vitality were observed (24 h). The morphological alterations of *A. fumigatus* biofilms treated with various ALA-PDT were shown in Fig. 5a, which included plain and three-dimensional views. The biofilms of *A. fumigatus* were treated with ALA alone, LED alone, ALA-PDT1 (ALA + LED [50 J/cm^2^]), ALA-PDT2 (ALA + LED [100 J/cm^2^]), and ALA-PDT3 (ALA + LED [200 J/cm^2^]). **b** After 24 h of culture, CLSM was used to evaluate biofilm thickness under various ALA-PDT treatments. Three different experiments' thickness averages are displayed. Biofilm thickness was lower in the ALA-PDT groups than in the control group. **c** The ALA-PDT treatment's inhibitory efficacy on *A. fumigatus* biofilm development was determined using the XTT test with various treatments, as shown. * *p* < 0.05 is a significant difference. Scale bar is 100 μm

To further understand the efficacy of this technique for biofilm eradication, the effect of ALA-PDT therapy on biofilm development in the early (4 h) and late (24 h) phases was studied. As previously stated, we employed six experimental groups for treatment and used CLSM to investigate the morphological effects (Figs. 4, 5).

The conidia concentration was 572.2 ± 13 cells/mm^2^ in the control group, 512.3 ± 28 cells/mm^2^ in the ALA alone group, 515.2 ± 31 cells/mm^2^ in the LED alone group, 422 ± 28 cells/mm^2^ in the ALA-PDT1 group, 287 ± 36 cells/mm^2^ in the ALA-PDT2 group, and 152 ± 10 cells/mm^2^ in the ALA-PDT3 group in the early stages of biofilm formation (4 h) (Fig. 4b). The conidia concentrations in the ALA-PDT groups were substantially lower than the control group (*p* < 0.05). Furthermore, the conidia adhesion rate in the control group was substantially higher than that in the ALA-PDT group. The conidia adhesion rate in the ALA-PDT3 group was likewise considerably lower than that in the ALA-PDT2 (*p* < 0.05) or ALA-PDT1 (*p* < 0.05) groups among the ALA-PDT groups. When compared to the control group, the adhesion rate in the ALA-PDT1 group reduced by 26.2 percent, 50.2 percent in the ALA-PDT2 group, and 73.4 percent in the ALA-PDT3 group at 4 h (Fig. 4c). At the same time, the ALA-PDT intervention was done on *A. fumigatus* cells that were in a planktonic state. The OD values at 490 nm of the control group were significantly higher than those of the ALA-PDT group when using XTT to measure changes in viability (Fig. 4d).

Biofilm structural organization was significantly disturbed when treated with ALA-PDT compared to the control. The more intense the light, the more terrible the commotion (Fig. 5). The drop in biofilm thickness mirrored the stated circumstance (Fig. 5b). The biofilm thickness in the control group was 195.3 ± 13.5 μm, 193.5 ± 10.2 μm in the ALA alone group, 198.4 ± 14.1 μm in the LED alone group, 175.4 ± 9.3 μm in the ALA-PDT1 group, 146.5 ± 3.6 μm in the ALA-PDT2 group, and 125.7 ± 3.2 μm in the ALA-PDT3 group at 24 h following ALA-PDT treatment. Biofilm thickness was lower in the ALA-PDT2 and ALA-PDT3 groups than in the control group. The biofilm thickness in the ALA-PDT3 group was substantially smaller than that in the ALA-PDT2 (*p* < 0.05) or ALA-PDT1 (*p* < 0.05) groups among the ALA-PDT groups.

We chose the late phases of biofilm development for observation following ALA-PDT therapy to better understand the influence of ALA-PDT on biofilm viability. At the late phases of biofilm, the OD value of the control group was substantially greater than that of the ALA-PDT groups (*p* < 0.05). The ALA-PDT3 group had a considerably lower OD value than the ALA-PDT2 (*p* < 0.05) or ALA-PDT1 (*p* < 0.05) groups among the ALA-PDT groups (Fig. 5c). To verify the conclusion that ALA-PDT inhibits biofilm viability and mature explicitly, we used 3 *A. fumigatus* (A1f, 02436, 02439) for further study. The structure of the biofilms were damaged and become loose after the ALA-PDT treatment as compared to the blank control. The ALA-PDT treatment's inhibitory efficacy on *A. fumigatus* biofilm development was determined by using XTT, and the activity of the biofilms was markedly suppressed (Supplementary Fig. 2).

## Discussion

*A. fumigatus* is a widespread opportunistic bacterium that causes infections in the dermis, bloodstream, and lungs in premature neonates and highly immunocompromised individuals [29–31]. A substantial death rate for *A. fumigatus* cutaneous infection has already been documented [1, 32], particularly in premature newborns. In PCA patients, drug resistance is a common reason of therapy failure. The establishment of a fungal biofilm results in a high level of antibiotic resistance. According to several studies, ALA-PDT has a strong inhibitory impact on microbial biofilms. There are currently no studies using ALA-PDT to target *A. fumigatus* biofilm.

Optical microscope (OM) [33], transmission electron microscopy (TEM) [34, 35], atomic force microscope (AFM) [36], and CLSM [37, 38] are some of the morphological study methods typically utilized in biofilm. CLSM was chosen as the approach for seeing changes in biofilm structure in our study because it is the only technology that can monitor the 3D structure of biofilm without harming it. Furthermore, we used XTT for biofilm activity detection since it is extensively utilized in microbial biofilm studies [39].

We were able to successfully create an in vitro *A. fumigatus* biofilm model in this investigation. The initial phase in biofilm development, adhesion of *A. fumigatus* conidia, is closely linked to biofilm construction success [40]. Biofilm development will be ineffective if the conidia concentration is incorrect [6]. Eilidh's research looked at the influence of different conidial densities, ranging from1 × 10^6^ conidia ml^−1^, on biofilm development. The best acceptable conidial density was found to be 1 × 10^5^ conidia ml^−1^. Biofilms generated by fewer planted conidia (1 × 10^4^ conidia ml^−1^) had a thicker but looser structure. This means that biofilm may be removed more readily by mechanical forces and so is not repeatable. The thinner biofilm produced less biomass than the conidial concentrations of 1 × 10^5^ conidia ml^−1^ at higher conidial concentrations (1 × 10^6^conidia ml^−1^). As a result, we look at the impact of ALA-PDT not only on the mature structure of biofilm, but also on the early stage of conidia attachment, which is linked to biofilm development.

To investigate the effect of ALA-PDT on *A. fumigatus* biofilm killing, we first looked at the effect of ALA or LED alone on biofilm. The viability of the *A. fumigatus* biofilm was not affected by ALA or LED alone, according to our findings. This is in line with earlier *Candida* biofilm findings [27]. It has been demonstrated that following treatment with three different doses of ALA (5 mM, 15 mM and 30 mM), there was no significant change in *C. albicans* biofilm viability between the experimental and control groups. In *Staphylococcus Aureus*, the fact that photosensitizers or LEDs alone have no effect on the survival of microbial biofilms has been validated [41]. In summary, the ideal ALA concentration was determined to be 15 mM.

The regulation of biofilm growth relies heavily on conidia adherence. The rate of conidia adhesion dropped dramatically after 4 h of treatment with ALA-PDT, according to our findings. This inhibitory effect is consistent with previous *C. albicans* research [27]. After treatment with ALA-PDT, they discovered a substantial reduction in the expression of *C. albicans* genes involved in adhesion and biofilm formation (ALS1 and HPW1) [42]. As a result, ALA-PDT may result in a large reduction in the expression of *A. fumigatus* adhesion genes.

The three-dimensional structure of the *A. fumigatus* biofilm might be a possible antimicrobial drug defense mechanism [43]. Our findings indicate that while ALA-PDT therapy can disturb the maintenance of mature biofilms, it cannot completely eliminate it. Part of the reason might be due to the biofilm’s three-dimensional structure. Another reason might be because suspended cell conidia came into touch with and were absorbed by the ALA solution more than the biofilm's three-dimensional structure. Several investigations have also confirmed this. They discovered that ALA-PDT therapy inhibits planktonic pathogens more effectively than biofilm treatment [44]. Furthermore, we discovered that early-stage ALA-PDT therapy inhibited biofilm development more effectively than late-stage ALA-PDT treatment. As a result of our research, we believe it is critical to start ALA-PDT therapy as soon as possible following a CIA diagnosis.

## Conclusions

Overall, our findings show that ALA-PDT inhibits *A. fumigatus* biofilm formation. ALA-PDT has the potential to be a critical tool in the treatment of CIA, according to our findings. In the future, more in vitro and in vivo investigations, as well as clinical trials, will be needed to verify these findings.

### Supplementary Information

Below is the link to the electronic supplementary material.

Supplementary file1 (TIF 14217 KB)

Supplementary file2 (TIF 17342 KB)

Supplementary file3 (DOCX 15 KB)

## Data Availability

All data generated or analyzed during this study are included in this published article.

## List of abbreviations

ALA: 5-Aminolevulinic acid
PDT: Photodynamic therapy
CFU: Colony-forming units
XTT: 2,3-Bis(2-methoxy-4-nitro-5-sulfo-phenyl)-2Htetrazolium-5-carboxanilide
LED: Light-emitting Diode
SDA: Sabouraud dextrose agar
CLSM: Confocal laser scanning microscope
PBS: Phosphate-buffered saline
PCA: Primary cutaneous aspergillosis
