# Supplementary figures and images for: Effect of 5-Aminolevulinic Acid Photodynamic Therapy on Aspergillus fumigatus Biofilms in Vitro
Source: Curr Microbiol. 2023 Sep 2;80(10):334. doi: 10.1007/s00284-023-03351-8 (PMC10474982; doi:10.1007/s00284-023-03351-8)

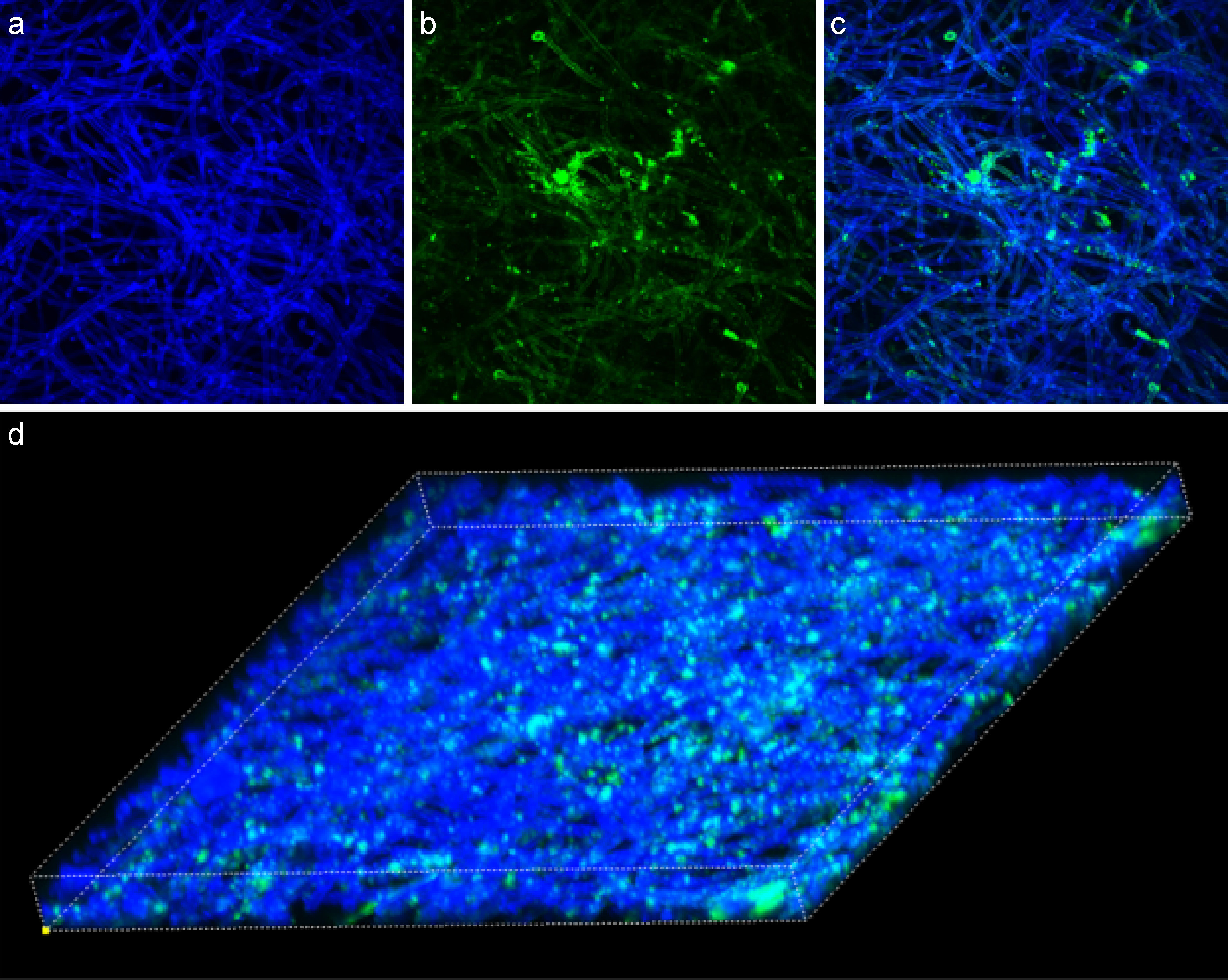

Supplement: Supplementary file 1 — Supplementary file1 (TIF 14217 KB) [file 284_2023_3351_MOESM1_ESM.tif]

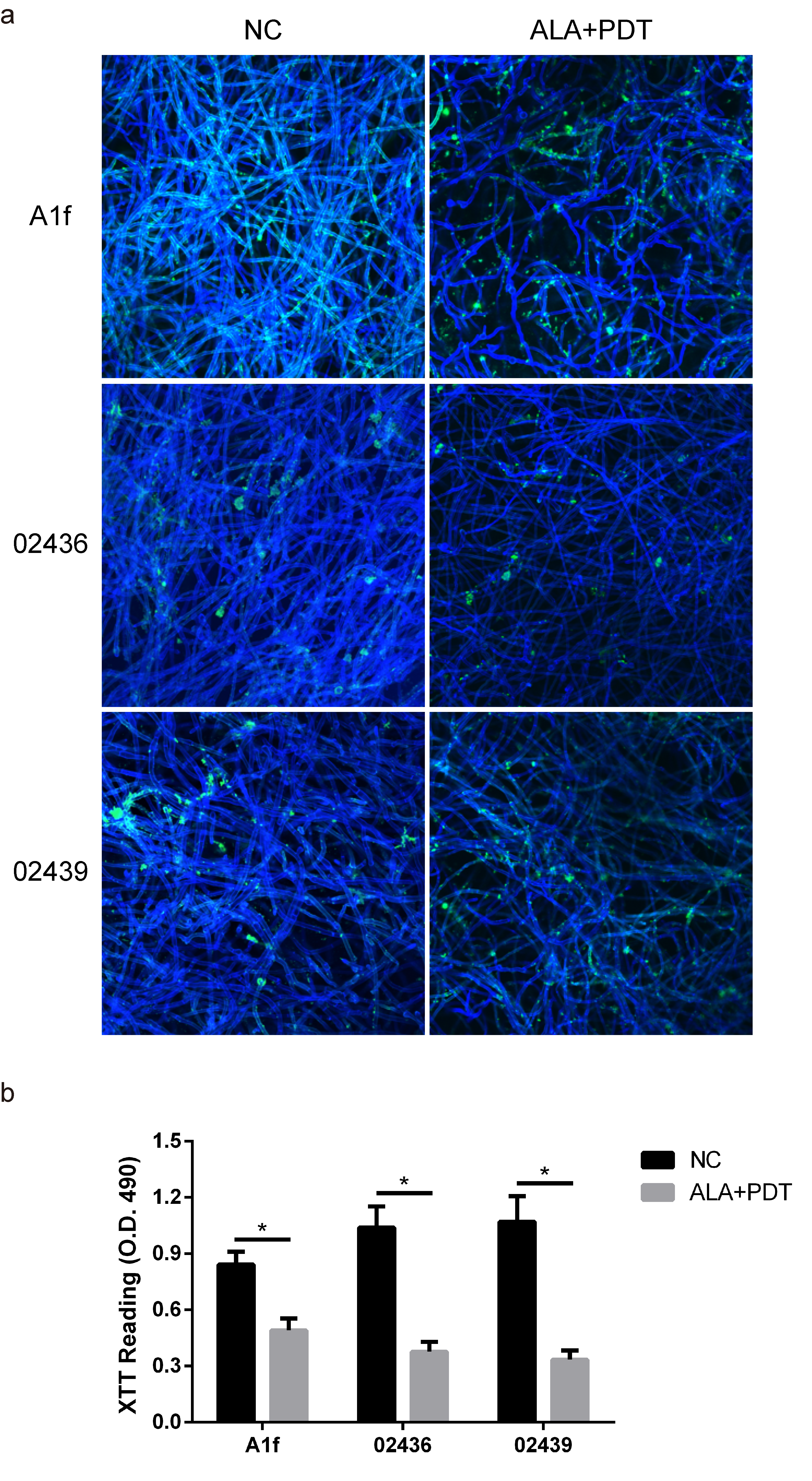

Supplement: Supplementary file 2 — Supplementary file2 (TIF 17342 KB) [file 284_2023_3351_MOESM2_ESM.tif]
