# Supplementary material for: Effect of 5-Aminolevulinic Acid Photodynamic Therapy on Aspergillus fumigatus Biofilms in Vitro
Source: Curr Microbiol. 2023 Sep 2;80(10):334. doi: 10.1007/s00284-023-03351-8 (PMC10474982; doi:10.1007/s00284-023-03351-8)
Supplement: Supplementary file 3 — Supplementary file3 (DOCX 15 KB) [file 284_2023_3351_MOESM3_ESM.docx]

**Article title:** Effect of 5-aminolevulinic acid photodynamic therapy on *Aspergillus fumigatus* biofilms *in vitro*

**Journal name:** Current Microbiology

Zhimin Duan^2^, Jianbo Tong^3^, Nana Zheng^2^, Rong Zeng^2,^ *, Yuzhen Liu^1,^ *, Min Li^2, 4,^ *

^1^ Department of dermatology, Nanjing Jiangning Hospital, Nanjing 211100, Jiangsu, China.

^2^ Hospital for Skin Diseases (Institute of Dermatology), Jiangsu Key Laboratory of Molecular Biology for Skin Diseases and STIs, Chinese Academy of Medical Sciences and Peking Union Medical College, Nanjing 210042, Jiangsu, China.

^3^ Department of Dermatology, the First Affiliated Hospital of Nanchang University; Institute of Dermatology, Jiangxi Academy of Clinical Medical Sciences; No. 17 Yongwaizheng Street, Nanchang 330001, Jiangxi, China.

^4^ Center for Global Health, School of Public Health, Nanjing Medical University, Nanjing 211166, Jiangsu, China.

Zhimin Duan and Jianbo Tong contributed to this article equally.

*** Corresponding author:** Rong Zeng, Yuzhen Liu and Min Li

**Rong Zeng**

Institutional email address: zengrong@pumcderm.cams.cn

**Yuzhen Liu**

Institutional email address: liuyuzhen0409@student.pumc.edu.cn

**Min Li**

Institutional email address: [limin@pumcderm.cams.cn](mailto:limin@pumcderm.cams.cn)

**Supplementary fig. 1** The formation of *Aspergillus fumigatus* biofilms was captured by CLSM at 24 hours. Biofilms were stained with Concanavalin A Alexa fluor 488 (green, a) and Calcofluor white (blue, b). The merge image highlights the production of extracellular polysaccharides from biofilms (c). Software was used to create a three-dimensional picture of the biofilm at 24 hours (d). FUN-1 was used to dye the cells. The original magnification was ×400.

**Supplementary fig. 2** After ALA-PDT treatment, changes in biofilm morphology and vitality of other *A. fumigatus* were observed. The morphological alterations of 3 *A. fumigatus* (A1f, 02436, 02439) biofilms treated with ALA-PDT were shown, and the structure of the biofilms were damaged and become loose as compared to the blank control (a). The ALA-PDT treatment's inhibitory efficacy on *A. fumigatus* biofilm development was determined using XTT with treatments (b), and the activity of the biofilms was markedly suppressed. * *p* <0.05 is a significant difference.
